# Supplementary material for: Glucose-Stimulated Calcium Dynamics in Beta Cells From Male C57BL/6J, C57BL/6N, and NMRI Mice: A Comparison of Activation, Activity, and Deactivation Properties in Tissue Slices
Source: Front Endocrinol (Lausanne). 2022 Mar 24;13:867663. doi: 10.3389/fendo.2022.867663 (PMC8988149; doi:10.3389/fendo.2022.867663)
Supplement: Supplementary file 1 [file DataSheet_1.docx]

Supplementary Material

# Supplementary Figures

**
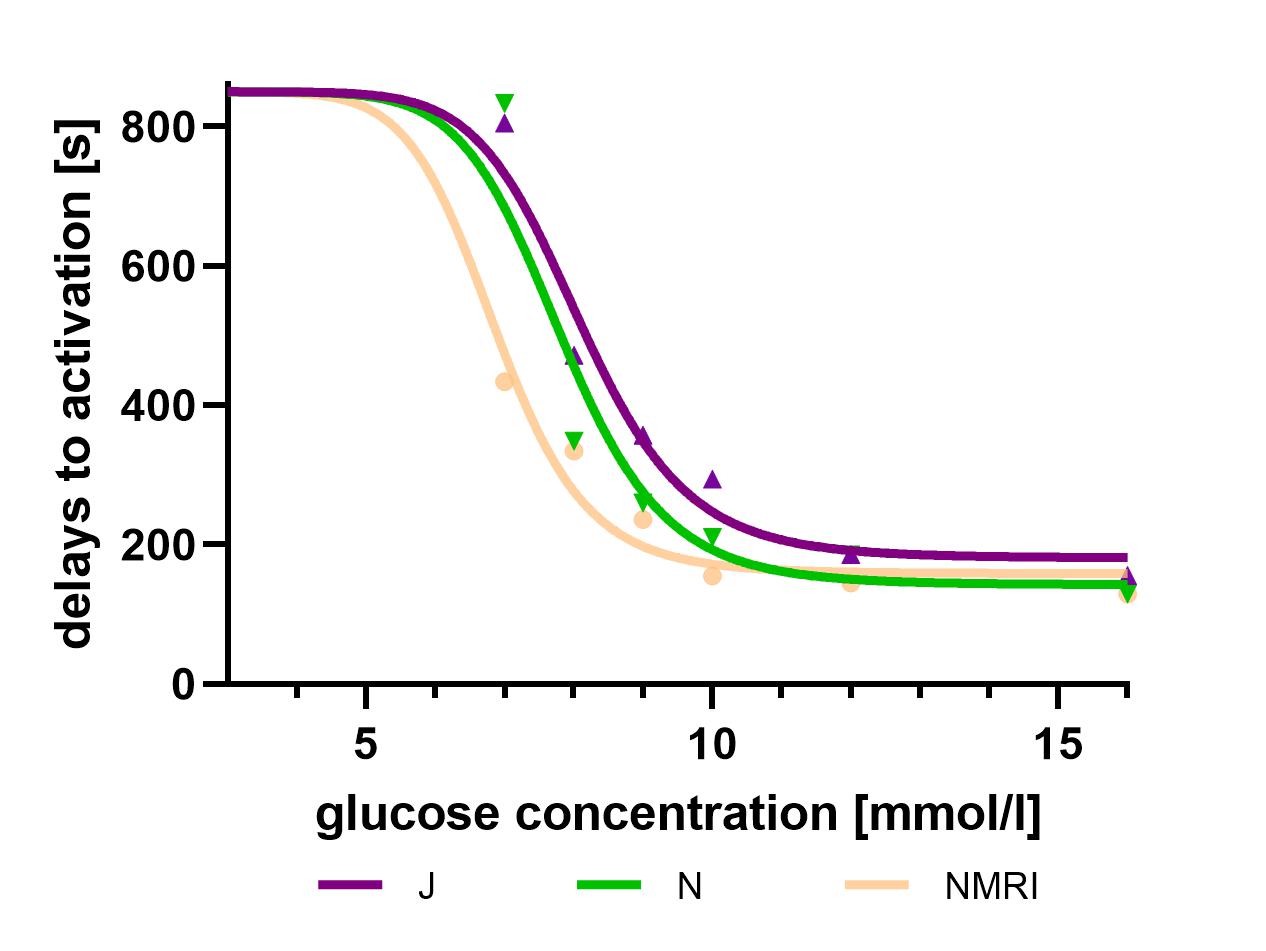
**

**Supplementary Figure 1.** **Activation delays of beta cells are left-shifted in NMRI mice compared to the C57BL/6J and C57BL/6N.** Fitted 4-parameter logistic regression model of delays in activation of beta cells in C57BL/6J, C57BL/6N and NMRI mice at 7-, 8-, 9-, 10-, 12-, and 16- mM glucose. Delays to activation in C57BL/6J (median 805, 472, 357, 294, 186, and 156 s), C57BL/6N (median 833, 349, 260, 210, 185, 129 s), and NMRI (median 434, 334, 236, 155, 145, and 129 s) at 7-, 8-, 9-, 10-, 12-, and 16-mM glucose, respectively. Pooled data (coded as C57BL/6J | C57BL/6N | NMRI) from the following number of cells/islets/pancreas preparations: 239/9/6| 643/13/6| 743/13/6 (7mM glucose), 370/10/7| 876/8/5| 730/12/6 (8 mM glucose), 657/11/7| 851/9/6| 1091/12/6 (9 mM glucose), 521/9/6| 756/9/5| 1078/10/6 (10 mM glucose), 681/11/7| 759/11/6| 904/10/5 (12 mM glucose), and 725/11/5| 703/8/5| 1061/11/6 (16 mM glucose).


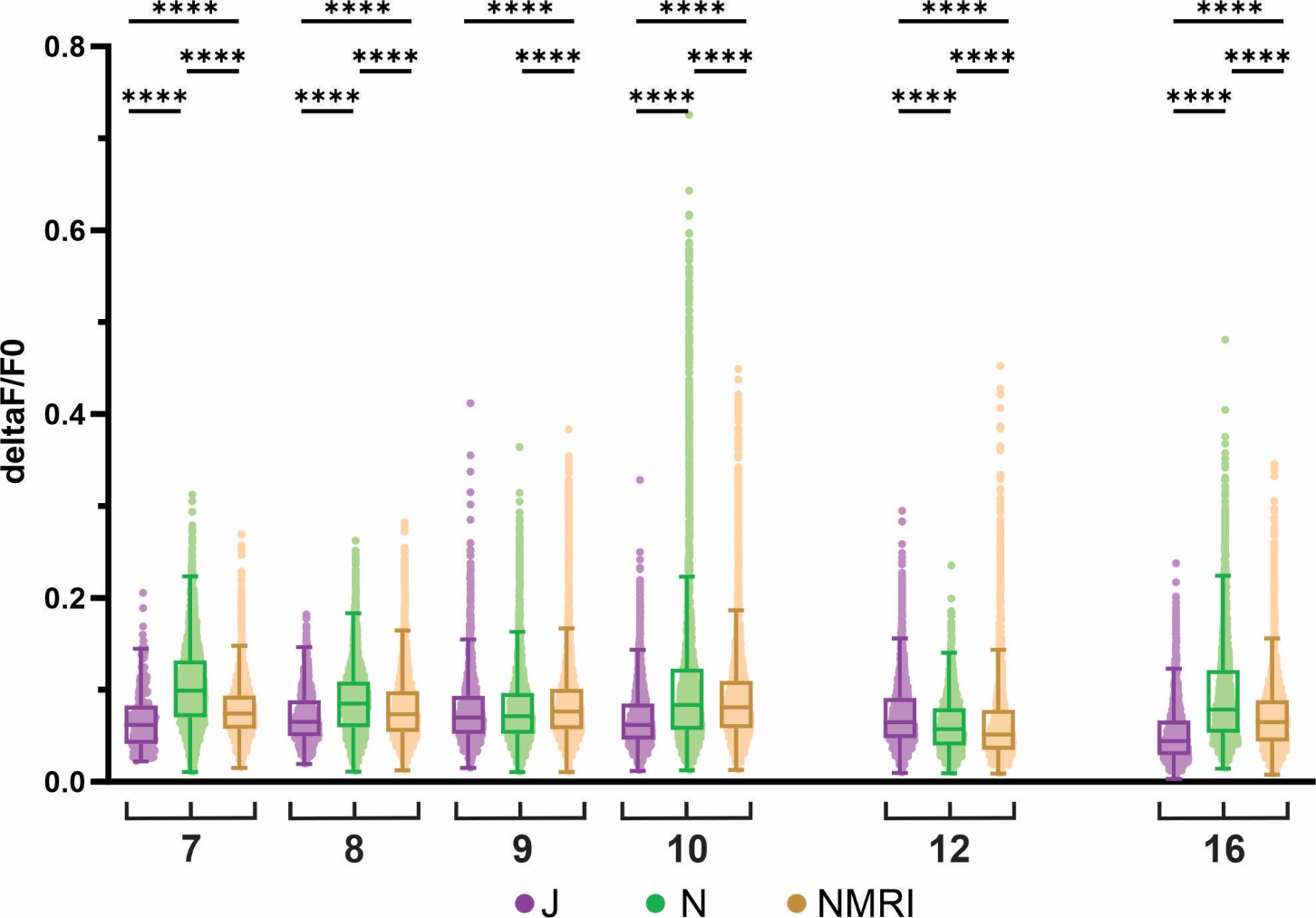


**Supplementary Figure 2. Amplitude of fast oscillations during the plateau phase of beta cell response to glucose in C57BL/6J, C57BL/6N, and NMRI mice.** Amplitude is expressed as ΔF/F_0_, i.e., change in fluorescence (ΔF) relative to the basal fluorescence (F_0_). X labels denote the tested glucose concentration. C57BL/6J: median 0.062, 0.065, 0.070, 0.062, 0.065, and 0.044; C57BL/6N: 0.099, 0.085, 0.071, 0.084, 0.057, and 0.079; NMRI: 0.074, 0.073, 0.076, 0.081, 0.051, and 0.065 in 7-, 8-, 9-, 10-, 12-, and 16-mM glucose, respectively. Pooled data (coded as C57BL/6J | C57BL/6N | NMRI) from the following number of cells/islets/pancreas preparations: 57/6/5| 325/11/6| 292/11/6 (7 mM glucose), 96/10/7| 671/8/5 381/10/5 (8 mM glucose), 246/8/7| 684/9/6| 574/10/6 (9 mM glucose), 250/8/5| 531/9/5| 677/9/6 (10 mM glucose), 322/10/7| 456/9/4| 310/7/5 (12 mM glucose), and 256/9/5| 414/7/5| 458/8/6 (16 mM glucose).


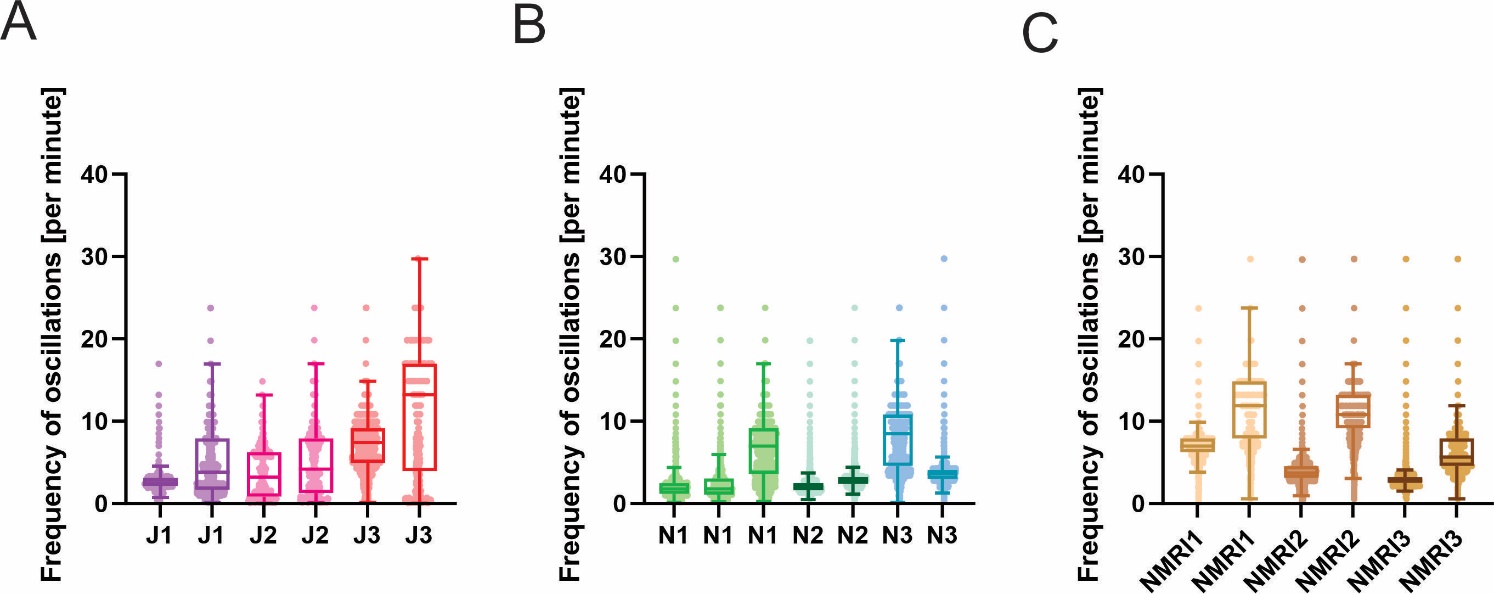


**Supplementary Figure 3. Beta cell oscillation frequencies shown for individual islets during stimulation with 10 mM glucose.** A subset of data from Figure 3 is shown for experiments in which >1 islet was recorded from one animal. X-axis labels define the animal from which the recording was obtained (letter depicting the strain: J for C57BL6/J, N for C57BL6/N, and N for NMRI, and the number the consecutive animal tag). Data points represent frequency derived from interburst intervals, and overlayed is the box plot representation of the data. Median values (from left to right on the respective panels): NMRI: 7.0, 11.9, 3.7, 10.8, 2.9, and 5.7 min^-1^; C57BL6/J: 2.5, 3.8, 3.2, 4.2, 7.4, and 13.2 min^-1^; C57BL6/N: 1.8, 1.8, 7.0, 1.9, 2.8, 8.5, and 3.6 min^-1^. Pooled data from the following number of cells (from left to right on the respective panels): NMRI: 56, 126, 138, 127, 104, and 126; C57BL6/J: 15, 92, 33, 36, 94, and 78; C57BL6/N: 68, 53, 56, 46, 32, 31, and 134.
